# Mental health implications of COVID-19 pandemic and its response in India

**DOI:** 10.1177/0020764020950769

**Published:** 2020-09-01

**Authors:** Adrija Roy, Arvind Kumar Singh, Shree Mishra, Aravinda Chinnadurai, Arun Mitra, Ojaswini Bakshi

**Affiliations:** 1 Department of Community Medicine and Family Medicine, All India Institute of Medical Sciences, Bhubaneswar, Odisha, India; 2 Department of Psychiatry, All India Institute of Medical Sciences, Bhubaneswar, Odisha, India; 3 Independent Researcher, Bhubaneswar, Odisha, India; 4 Independent Researcher, Kalyani, West Bengal, India

**Keywords:** Mental health, COVID-19, pandemic, stress, anxiety, depression

## Abstract

**Introduction:**

Mental health concerns and treatment usually take a backseat when the limited resources are geared for pandemic containment. In this global humanitarian crisis of the COVID-19 pandemic, mental health issues have been reported from all over the world.

**Objectives:**

In this study, we attempt to review the prevailing mental health issues during the COVID-19 pandemic through global experiences, and reactive strategies established in mental health care with special reference to the Indian context. By performing a rapid synthesis of available evidence, we aim to propose a conceptual and recommendation framework for mental health issues during the COVID-19 pandemic.

**Methods:**

A search of the PubMed electronic database and google scholar were undertaken using the search terms ‘novel coronavirus’, ‘COVID-19’, ‘nCoV’, SARS-CoV-2, ‘mental health’, ‘psychiatry’, ‘psychology’, ‘anxiety’, ‘depression’ and ‘stress’ in various permutations and combinations. Published journals, magazines and newspaper articles, official webpages and independent websites of various institutions and non-government organizations, verified social media portals were compiled.

**Results:**

The major mental health issues reported were stress, anxiety, depression, insomnia, denial, anger and fear. Children and older people, frontline workers, people with existing mental health illnesses were among the vulnerable in this context. COVID-19 related suicides have also been increasingly common. Globally, measures have been taken to address mental health issues through the use of guidelines and intervention strategies. The role of social media has also been immense in this context. State-specific intervention strategies, telepsychiatry consultations, toll free number specific for psychological and behavioral issues have been issued by the Government of India.

**Conclusion:**

Keeping a positive approach, developing vulnerable-group-specific need-based interventions with proper risk communication strategies and keeping at par with the evolving epidemiology of COVID-19 would be instrumental in guiding the planning and prioritization of mental health care resources to serve the most vulnerable.

## Background

The World Health Organisation (WHO) declared COVID-19 as a Public Health Emergency of International Concern (PHEIC) on 30 January 2020 (*Coronavirus (COVID-19) events as they happen*, 2020.) COVID-19 Pandemic has reached a level of a humanitarian crisis with over 6 million confirmed cases and 350,000 deaths reported globally to date (Up to 31st May 2020). PHEICs can pose a significant mental health risk to communities (Davis et al., 2010) especially in developing countries, where the risk is further precipitated by suboptimal socio-economic determinants (*COVID-19: impact could cause equivalent of 195 million job losses, says ILO chief | | UN News*, 2020). The consequences of COVID-19 impacts not only the physical health and wellbeing but also the mental health, which can have a disastrous effect on the health system.

Mental health concerns and treatment usually take a backseat when the limited resources are geared for pandemic containment. History suggests that any infectious disease outbreak or pandemic brings with itself a major setback in the mental health front. In the case of the Ebola outbreak in the year 2014, symptoms of Post-Traumatic Stress Disorder (PTSD) and anxiety-depression were more prevalent even after 1 year of Ebola response (Jalloh et al., 2018). The global HIV pandemic also provides a similar picture. It has been found that the prevalence of mental illnesses in HIV-infected individuals is substantially higher than in the general population. (World Health Organization, 2008) The risk of PTSD in the aftermath of the pandemic can, therefore, be a huge challenge to the mental health system of the country. Since the healthcare system focuses majorly on emergency services, individuals suffering from substance abuse and dependency disorders may see deterioration in their mental health as a result of this pandemic. (Clay & Parker, 2020) The economic fallout and forecasted recession pertaining to ‘The Great Lockdown’ is feared to be the worst global economic crisis after ‘The Great Depression’ (The Great Lockdown: Worst Economic Downturn Since the Great Depression – IMF Blog, 2020). With many sectors seeing pay-outs and job losses across Europe and America, unemployment can rise to a record 14% in the USA which can worsen to 20% post-pandemic in the future. This can lead to increase in suicide rates among the economically vulnerable (*COVID-19: Man commits suicide after being quarantined in Madhya Pradesh | Deccan* Herald, 2020; COVID-19 suspect *jumps to death at quarantine facility in Greater Noida, magisterial inquiry ordered |* India News, 2020). Reports of stigmatization of front-line workers resulting from the fear of getting 2..32the infection from them have surfaced across the world leading to increased mental health illnesses, like anxiety and depression among them Government, professional organizations, civil society bodies and other relevant stakeholders have come up with various measures in the context of mental health in a short span of time. In this study, we attempt to review the prevailing mental health issues during the COVID-19 pandemic through global experiences, and reactive strategies established in mental health care with special reference to the Indian context. By performing a rapid synthesis of available evidence, we aim to propose a conceptual and recommendation framework for mental health issues during the COVID-19 pandemic.

## Methods

The current article reviews the existing literature on mental health issues and interventions relevant to the COVID-19 pandemic. A search of the PubMed electronic database and google scholar was undertaken using the search terms ‘novel coronavirus’, ‘COVID-19’, ‘nCoV’, SARS-CoV-2, ‘mental health’, ‘psychiatry’, ‘psychology’, ‘anxiety’, ‘depression’ and ‘stress’ in various permutations and combinations. A thorough search of all published journal articles, newspaper articles, magazine articles, webpages including World health Organisation, Ministry of Health and Family Welfare- Government of India (MOHFW), State governments and independent websites of various institutions) and non-government organizations, and verified social media portals including -Twitter, Youtube, Facebook, Whatsapp, etc., have been compiled after exclusion of fake and unverified updates. The authenticity of the social media updates has been ensured by thorough search and inclusion of only verified institutional/organisational social media pages and central and state government social media portals. Different combinations of keywords including geographical locations, the vulnerable population were also used for the search strategy. Review was limited to search output up to 31st May 2020.

After review, we synthesized the evidence into two broad headings that is, mental health issues during COVID-19 pandemic particularly in the context of some vulnerable groups and possible reasons thereof, interventions recommended so far at a global level and India. Based on the evidence synthesis, we have proposed a conceptual framework for mental health risk during COVID-19 pandemic and a recommendation framework with reference to Low- and Middle-Income Countries (LMIC) like India

## Results

### Mental health issues during COVID-19 pandemic

The major mental health issues that have been reported to have been associated with the COVID-19 pandemic are **stress, anxiety, depressive symptoms, insomnia, denial, anger and fear** globally. (Torales et al., 2020) Stress, anxiety and depression go hand in hand with the COVID-19 pandemic, results from studies done globally has shown the increasing prevalence of mental health disorders among various population groups (Ji et al., 2017; Mohindra et al., 2020; Xiao et al., 2020b). Historically, disease pandemics have been associated with grave psychological consequences. A recent article published in JAMA Psychiatry suggests that COVID-19 may lead to increased risk of suicide (Xiang et al., 2020). A recent study done in China reported 16.5% moderate to severe depressive symptoms; 28.8% moderate to severe anxiety symptoms; 8.1% moderate to severe stress due to COVID -19 (Wang et al., 2020). Similar impacts of COVID-19 on mental health has also been seen in other countries like Japan, Singapore and Iran (Rajkumar, 2020). The grief and depression resulting from loss of a loved one, anxiety and panic due to uncertain future and financial turmoil may lead individuals to resort to these extreme measures. Reports of COVID-19 related suicides have been increasingly common in the world news. India is also not immune to this phenomenon. Cases of COVID-19 related suicide have been reported from Maharashtra, Uttar Pradesh, Assam, Kerala (Cullen et al., 2020; *Coronavirus in India: Suspected Covid-19 patient who committed suicide in UP hospital tests negative - India News*, 2020; *Anxiety over COVID-19 leads to Phagwara woman’s suicide : The Tribune India*, 2020). An Indian newspaper article published in May 2020 revealed that, Suicide was the leading cause for over 300 ‘noncoronavirus deaths’ reported in India due to distress triggered by the nationwide lockdown*(‘Suicides due to lockdown: Suicide leading cause for over 300 lockdown deaths in India, says study,’ 2020)*. Reports of suicide of healthcare workers, migrant labourers and those in quarantine centres have been frequenting in the news and media ever since the pandemic started to change the lives of people. Though some newspaper articles and webpages and researchers have reported deaths during the pandemic apart from the COVID-19 (which includes deaths due to mental health disorders, suicide, starvation, accidents etc.) (Thejesh, 2020; A Different Tragedy Strikes Kerala During COVID-19 Lockdown Due to Non-Availability of Alcohol, 2020; Corona scare drives youth to suicide, third in UP, 2020; Coronavirus Lockdown: Unable To Care For Family, Uttar Pradesh Man Commits Suicide In Lakhimpur Kheri, Blames Lockdown, 2020; Hyderabad tippler commits suicide upset at not getting liquor during lockdown- The New Indian Express, 2020; Two Covid-19 patients commit suicide at Chennai hospitals within 24 hours - India News, 2020), a huge area of ‘*Non COVID–19’* related deaths remain to be explored.

Strict lockdown laws, social distancing, restrictions in movement could result in increased screen time. Constant misinformation in social media portals may result in a state of panic and anxiety, often resulting in depression eventually. Findings of a study done in Shanghai, China show a high prevalence of mental health problems, which positively associated with frequent social media exposure during the COVID-19 outbreak (Gao et al., 2020).

Summary of studies reporting mental health effects of COVID 19 pandemic and previous pandemics are summarized in Table 1.

**Table 1.** Studies on mental health implications of different outbreaks, epidemics and pandemics globally.

Authors	Study design/ Methodology/Type of article	Study tools	Country	Study Population	Disaster/ Pandemic/ Epidemics	Results
Wang et al., 2020	Online survey	Depression, Anxiety and Stress Scale (DASS-21); Impact of Event Scale-Revised (IES-R)	China	General population (*n* = 1210)	COVID-19 Pandemic	16.5% moderate to severe depressive symptoms; 28.8% moderate to severe anxiety symptoms; 8.1% moderate to severe stress
Xiao et al., 2020a	Cross-sectional study, self-rated questionnaire	Self-Rating Anxiety Scale (SAS); General Self-Efficiency Scale (SES); Stanford Acute Stress Reaction Questionnaire (SASR); Pittsburgh Sleep Quality Index (PSQI); Social Support Rate Scale (SSRS)	China	Medical staff treating patients with COVID-19 (*n* = 180)	COVID-19 Pandemic	Mean anxiety scores 55.3 ± 14.2; anxiety positively correlated with stress and negatively with sleep quality, social support and self-efficiency (*p* < .05, all correlations)
Xiao et al., 2020b	Cross-sectional, self-rated questionnaire	Self-Rating Anxiety Scale (SAS); Stanford Acute Stress Reaction Questionnaire (SASR); Pittsburgh Sleep Quality Index (PSQI); Personal Social Capital Scale (PSCI-16)	China	Individuals in self-isolation for 14 days (*n* = 170)	COVID-19 Pandemic	Mean anxiety score 55.4 ± 14.3; Anxiety positively correlated with stress and negatively with sleep quality and social capital; social capital positively correlated with sleep quality. (*p* < .05, all correlations)
Li et al., 2020	Cross-sectional, self-rated survey using a mobile app	Chinese version of the Vicarious Traumatization Scale	China	General public (*n* = 214); front-line nurses (*n* = 234); non-front-line nurse (*n* = 292)	COVID-19 Pandemic	Traumatization related to COVID-19 higher among non-front line than front-line nurses (*p* < .001); traumatization among the general public higher than for front-line nurses (*p* < .005) but not non-front-line nurses
Mohindra et al. (2020)	Qualitative analysis	Interviews with health care professionals	India	Frontline health care providers (HP) of Tertiary hospital in North India involved in the care of patients with COVID-19 or suspected COVID-19	COVID-19 Pandemic	Following are the main themes identified for mental health promotion of HP: 1. Positive Motivational factors a. Intellectual b. Emotional 2. Negatives, frustrations associated with patient care 3. Personal fears and annoyances experienced by doctors
Banerjee, 2020	Letter to Editor	–	–	Geriatric	COVID-19	Key points declared • Elderly are most vulnerable for severity and mortality. • Elderly are most susceptible to mental health problems related to such pandemics. • Special care needs to be taken for geriatric mental health during such crisis. • make
Golberstein et al., 2019	Viewpoint	–	–	Children	COVID-19	Concluding statement- ‘COVID-19 will have major repercussions for children and adolescents’ health and well-being. Timely action can help lessen the effects and improve long-term capacities for mental health services’.
Andrew Secor et al., 2020	Cross sectional study	Patient Health Questionnaire-9 (PHQ-9) depression scores and Generalised Anxiety Disorder-7 (GAD-7) scores.	Liberia, Sierra Leone and Guinea	Ebola Virus Disease Survivors	Ebola Virus Disease Epidemic	Prevalence and severity of depression and anxiety varied across the three countries. Sierra Leone had the highest prevalence of depression (22%), 20.2% in Liberia and 13.0% in Guinea. Sierra Leone also showed the highest prevalence of anxiety, with 10.7% (GAD-7 score ⩾10), compared with 9.9% in Liberia and 4.2% in Guinea. More than 1 in 10 respondents reported ideation of self-harm or suicide (range: 19.4% in Sierra Leone to 10.4% in Guinea).
Ji et al., 2017	Cross sectional study	Symptoms Checklist 90-items, Revised (SCL-90-R)	Sierra Leone	161 participants including Sierra Leone (SL) medical staff (*n* = 59), SL logistic staff (*n* = 21), SL medical students (*n* = 22), and Chinese medical staff (*n* = 41), the other group consisted of 18 EVD survivors.	Ebola Virus Disease Epidemic	The order of total general severity index (GSI) scores from high to low was EVD survivors, SL medical staff, SL logistic staff, SL medical students, and Chinese medical staff. There were 5 dimensions (obsession-compulsion, anxiety, hostility, phobic anxiety, and paranoid ideation) extremely high in EVD survivors. GSI were associated with university education negatively.
IR Cabello et al. 2020	Systematic Review	–	Worldwide (59% from Asia)	Healthcare workers	Viral Infectious diseases 69%- SARS outbreak 2003	The pooled prevalence was higher for anxiety (45%) followed by depression (38%), acute stress disorder (31%) burnout (29) and post-traumatic stress disorder (19%).

### Mental health implications in specific population groups

#### People with pre-existing mental health illness

It is known that at the rise of an epidemic, generally, people with pre-existing mental health conditions are among the most affected (Chatterjee et al., 2020). The reasons include social stigmatization, risk of infection, low priority to morbidities of mental health etc. These coupled with cognitive impairment, little awareness of risk and diminished efforts regarding personal protection in patients, as well as confined conditions in psychiatric wards could add to the vulnerability of individuals with presenting mental health illnesses during the COVID-19 pandemic (Yao et al., 2020). Discrimination and fear of social isolation due to social distancing worsened by the effects of strict rules of lockdown could add to the cause of their vulnerability. The resulting emotional responses, leading to triggering, relapse or worsening of pre-existing mental health conditions could be another result of the effects of the COVID-19 pandemic. Wandering mentally ill people are at major risk of contracting illness secondary to compromised immune status. Relapse and exacerbation of severe mental health conditions secondary to lockdown and unavailability of psychotropics in rural pharmacies can also pose a hurdle to the health care system.

#### Frontline workers

The frontline workers including doctors, nurses, community health workers, sanitation workers, policemen, and other volunteers across the world are in an entirely unprecedented situation, having to make impossible decisions and work under extreme pressures. Working under stressful conditions with scarce resources affect not just their personal and family life, but also place them in a situation of moral injury, causing mental health problems. These symptoms can contribute to the development of mental health difficulties, including depression, post-traumatic stress disorder, and even suicidal ideation (Cheng et al., 2004; Duan & Zhu, 2020; Greenberg et al., 2020; Litz et al., 2009; Williamson et al., 2018). Apart from being at high risk of infection, front line healthcare workers including doctors are subject to stigma by community and neighborhoods. Many instances of eviction and harassment from house owners, violence on duties against doctors at the workplace, social isolation, and discrimination have been reported.

#### Children and older people

The sudden and drastic changes in the day to day routine can be extremely confusing and difficult to cope with the children, geriatric, and quarantined individuals. Closure of schools, recreational outdoor activities, not meeting their peers could take a toll on the mental health of the children. The geriatric population in India has been identified as a vulnerable group to COVID-19. Over 50% of those more than 60 years have at least 1 comorbidity putting them at a much higher risk. The psychological impacts of these populations can include anxiety and feel stressed or angry. Mental health impact can be particularly difficult for older people who are already experiencing cognitive decline, dementia, social isolation, and loneliness. Also, the progression of the disease tends to be more severe in the case of elderlies resulting in higher mortality (MOHFW, 2020).

Probable reasons (both evidence-based and theoretical possibilities) of mental health effects during COVID-19 pandemic among specific vulnerable population groups are summarized in Table 3.

### Intervention strategies for mental health issues during COVID-19

#### Global context

A nationwide survey of psychological distress among Chinese people in the COVID-19 epidemic suggests the following recommendations for future interventions: (a) increased attention needs to be paid to vulnerable groups such as the young, the elderly, women and migrant workers; (b) the availability and accessibility of medical resources and the public health service system should be further strengthened and improved, with lessons from the management of the COVID-19 epidemic; (c) nationwide strategic planning and coordination for psychological first aid during major disasters, potentially delivered through telemedicine, should be established and (d) comprehensive crisis prevention and intervention system should be built including stable surveillance and monitoring systems, screening, referral, and targeted interventions should be built to reduce psychological distress and prevent further mental health problems.

On 27 January 2020, the National Health Commission in Mainland China issued the first comprehensive guidelines on emergency psychological crisis intervention in individuals who were affected by COVID-19; 19 the emphasis was on the delivery of mental health support services to patients and HCW by multidisciplinary teams that consisted of mental health professionals (Ho, 2020). In Singapore, the Ministry of Health, have kept the public abreast of the progress of the outbreak with regular news broadcasts and announcements on social media. Social media channels have also been set up by the state to curb the spread of false information and ‘fake news’ (Ho, 2020). Some interventions used by China, where the pandemic was first reported were, ‘Expert-teacher-coach’ intervention, frequently issuing guidelines for ‘Emergency Psychological Assistance’ by National Health Commission of China, applications like ‘WE-CHAT’ based survey program, online education using ‘WE-CHAT’, ‘WEIBO’ and ‘TIKTOK’. Artificial Intelligence-based ‘Tree hole rescue’ has also been utilized to combat mental health concerns and could be incorporated in other countries including India (Liu et al., 2020).

The Centre for Disease Control (CDC) advises parents to watch for changes in behaviour in their child. Since not all children and teens respond to stress in the same way a thorough and timely lookout for alert signs are important. Some common changes to watch for include the following (*Mental Health and Coping During COVID-19 | CDC*, 2020): that is, Excessive crying or irritation in younger children, Returning to behaviours they have outgrown (e.g. toileting accidents or bedwetting), Excessive worry or sadness, Unhealthy eating or sleeping habits, Irritability and ‘acting out’ behaviors in teens, Poor school performance or avoiding school, Difficulty with attention and concentration, Avoidance of activities enjoyed in the past, Unexplained headaches or body pain, Use of alcohol, tobacco or other drugs.

In Italy, many independent (mainly online) initiatives have been established to provide psychological and psychiatric support to health professionals and laypeople, such as the ‘NON SEI SOLO’ [‘YOU ARE NOT ALONE’] and ‘Resilienza COVID-19’ [‘Resilience COVID-19’] projects of Rome’s Fondazione Policlinico Universitario Agostino Gemelli (Sani et al., 2020).

When looking largely at a global scenario, the varied population profile and mental healthcare needs of different countries are different. Therefore the intervention strategies taken up by the other high-income countries may not necessarily be effective in the context of India or similar Low and Middle-Income Countries (LMICs).

#### Indian context

The mental health issues in the context of the COVID-19 pandemic in India is more complex due to large proportion of socially and economically vulnerable population (children, geriatric, migrant laborers, etc.), high burden of pre-existing mental illness (Murthy, 2017), constrained mental health services infrastructure (Cullen et al., 2020), less penetration of digital mental health solutions, and above all scare created due to tremendous misinformation on social media. Thus, interventions should also be specific and relevant to the circumstances in India. The MOHFW- GOI has issued a tollfree helpline number for ‘Behavioural Health’, The Psycho-Social toll-free helpline-08046110007 can be used by anyone needing mental health assistance during the COVID-19 pandemic. A list of videos, advisories and resource materials on coping stress during COVID, yoga and meditation advice, taking care of the mental health of vulnerable groups, etc. have been provided in the MOHFW-GOI web portal (*MoHFW | Home*, 2020).

The existing mental health-related initiatives include guidelines detailing about mental health and psychosocial considerations during the COVID-19 outbreak developed by the WHO Department of Mental Health and Substance Use, as a series of messages that can be used in communications to support mental and psychosocial well-being in different target groups during the outbreak (*Mental Health and Psychosocial Considerations During the COVID-19 Outbreak*, 2020). The Ministry of health and family welfare, Government of India has published IEC materials on mental health care of the elderly and children. It also has materials on understanding the lockdown situation, handling isolation, dealing with mental health issues after recovering from COVID-19 (*MoHFW | Home*, 2020). Various other portals and institutions like The National Institute of Mental Health and Neuro-Sciences (NIMHANS), All India Institute of Medical Sciences, Indian Psychiatric Society have taken up independent responsibilities to promote and manage mental health issues during the COVID-19 pandemic in the form of online services, telemedicine services, etc.

NIMHANS suggests that a ‘Psychological intervention medical team’ can be formed as a standalone team or be part of the general medical team attending to people affected by the pandemic. The staff should consist of psychiatrists, with clinical psychologists and psychiatric nurses participating and the teams should formulate interventions plans separately for different groups for example: (i) Confirmed cases who are hospitalised with severe symptoms (ii) Suspected cases and close contacts of confirmed cases (iii) People with mild symptoms who are in home quarantine (iv) Health care personnel working with people with COVID-19 (v) General public (Cullen et al., 2020) As it is, mental health alone is a global challenge in itself and the COVID-19 pandemic greatly escalated the mental health burden as well.

Another initiative by the GOI is the *Aarogya Setu* mobile application which is used to connect essential health services with the people of India in our combined fight against COVID-19. The app is aimed at augmenting the initiatives of the Government of India, particularly the Department of Health, in proactively reaching out to and informing the users of the app regarding risks, best practices, and relevant advisories about the containment of COVID-19.

For the frontline workers fighting against this global crisis, routine support activities should be made available and must efficiently incorporate and include a briefing on moral injuries. It should also focus on raising awareness of other causes of mental ill-health and what to look out for.

Apart from these, mental health interventions have been issued by different states, NGOs and organisational bodies, some of which are listed in Table 2.

**Table 2.** Some specific mental health interventions or initiatives taken by different states and institutional/ organizational bodies.

State/ Institution	Initiative	
Government of Maharashtra	The Maharashtra government launched two helpline numbers 1800120820050 and 18001024040.	This helpline number was started by the Mumbai municipal corporation in collaboration with Mpower, an initiative of the Aditya Birla Education Trust to deal with stress during the lockdown (*Mumbai: Helplines for mental health inundated with calls during coronavirus lockdown - India News*, 2020)
Government of Telangana	Telangana Government initiated mental health counselling through 108 helplines	The State Government has decided to utilise the 108 helpline for providing much-needed mental health counselling support to the general public during the ongoing lockdown (*Govt to use 108 helpline for mental health counselling- The New Indian Express*, 2020)
Government of Madhya Pradesh	Madhya Pradesh government has decided to revive the Department of Happiness initiative in the state	Under the initiative, the hospitals with COVID-19 patients will be provided with light entertainment, music, and films while the Happiness department which is also known as- Anand department will conduct necessary activities in association with social workers (*Coronavirus India: Madhya Pradesh Happiness Department To Reduce Stress Of COVID-19 Patients*, 2020)
Government of Kerala	Kudumbashree launches a special drive to help the elderly during the lockdown	A special initiative to ensure the well-being of elderly people in the state during the Covid-19 lockdown, by reaching out to families with a string of confidence-building measures. The thrust of the outreach programme is to take care of the mental health of the elderly and boost their confidence through appropriate IEC materials. (*Kudumbashree launches special drive to help elderly during lockdown- The New Indian Express*, 2020)
Government of Odisha	The Department of Health and Family Welfare, Odisha issued specific guidelines to take care of the vulnerable population groups Specific Telemedicine helpline numbers for mental health issues and continuum of healthcare	These guidelines are directed to be used as Information, Education and Communication materials to promote and protect the mental health of the general population as well as specific vulnerable groups of people (*Health Department*, 2020).
NIMHANS, Bengaluru	Govt announces Toll free number for Mental Health Issues with the Help of NIMHANS	The government has launched a toll-free helpline number – 08046110007 – for people who may face any mental health issue due to the ongoing countrywide lockdown to contain the spread of coronavirus. (*Government launches helpline for mental health issues during lockdown - The Economic Times*, 2020).
Institute of Mental Health in Hyderabad	With lockdown stressing out the mind, Hyderabad sets up an all-India helpline.	Notwithstanding the lockdown and travel restrictions, over 800 persons have reached out to the Institute of Mental Health at Erragadda in Hyderabad as outpatients in the last two weeks seeking treatment to various issues. About 170 have been admitted (*With lockdown stressing out the mind, Hyderabad sets up an all-India helpline | coronavirus outbreak News, The Indian Express*, 2020).
Tamil Nadu Psychology Association	Telepsychiatry counselling in Tamilnadu	Mastermind Foundation, a center for mental health based in Chennai, had brought together more than 60 psychologists from across India to offer counselling support, round-the-clock and in 11 languages (*Helpful numbers in these times - The Hindu*, 2020)

## Discussion

Previous research has revealed a profound and broad spectrum of psychological impact that outbreaks can inflict on people. We found in this review that stress, anxiety, depressive symptoms, insomnia, denial, anger and fear were the major mental health manifestations of the COVID 19 pandemic. Anecdotal evidences and newspaper report also suggest an increasing trend of suicide in the community, people with COVID 19, and people in quarantine and isolation.

Fear of disease can precipitate new psychiatric symptoms in people without mental illness, aggravate the condition of those with pre-existing mental illness and cause distress to the caregivers of affected individuals. Regardless of exposure, people may experience fear and anxiety regarding falling sick or dying, helplessness, or blame people who are ill, potentially triggering off a mental breakdown (Ho et al., 2020). Anxiety and fear related to an infection can lead to acts of discrimination. For example, People from Wuhan were targeted and blamed for the COVID-19 outbreak by other Chinese people and Chinese people have since been stigmatized internationally, for example, use of the term ‘China virus’ and the use of terms such as ‘Wuhan virus’ and the ‘New Yellow Peril’ by the media (Usher et al., 2020).

**Figure 1.**
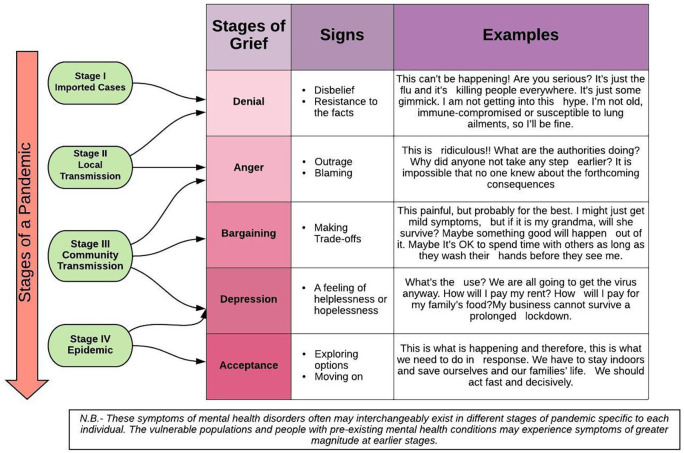
The stages of COVID-19 pandemic explained according to Kubler-Ross model of stages of grief.

The news of a pandemic is no less than a news of death and morbidity. In the case of COVID-19, we have tried to micro conceptualize based on the concept of ‘Breaking bad news’ and how the news of the pandemic was perceived globally could be very well classified according to stages of grief. Figure 1 shows how the stages of the COVID-19 pandemic could be placed into the Kubler-Ross stages of grief (*How to identify the stages of grief in COVID-19 messages - PR Daily*, 2020). The concept of crisis Communication is therefore what is needed in order to manage the mental health issues in the times of COVID-19 pandemic. With the world having witnessed about 7 pandemics over the last 100 years, the public health professionals across the globe have had the time to assess the impact such diseases have on human behaviour and communication. With no existing form of immunity against the pathogen and no availability of effective drugs or vaccines, behavioural actions (such as physical distancing and frequent handwashing) and risk communication becomes the first line of intervention.

**Figure 2.**
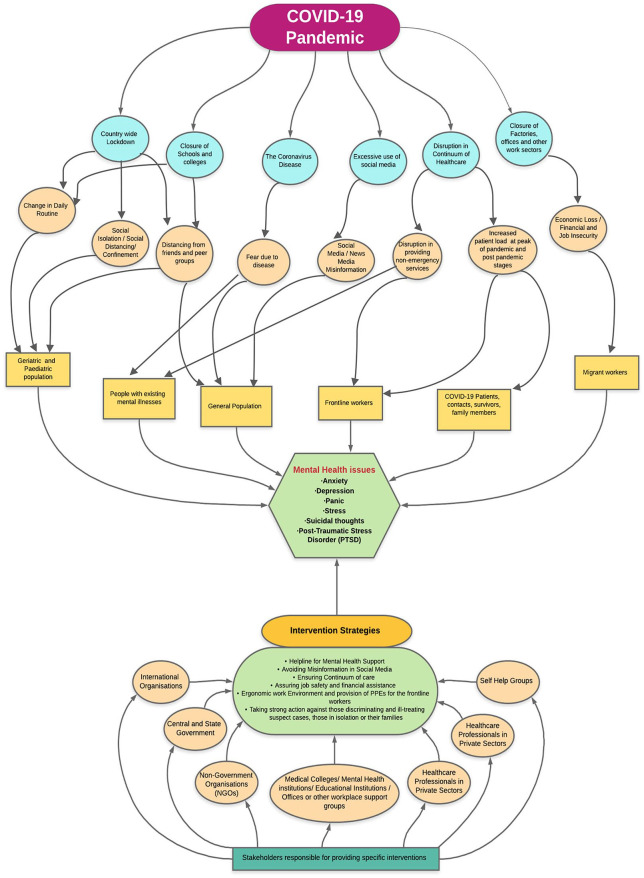
Conceptual framework of mental health issues during COVID-19 pandemic, its risk factors or causes; and some recommended intervention strategies.

We developed a conceptual framework both on the existing evidence and well as theoretical plausibility of mental health implication of COVID 19 pandemic and its response (Figure 2) While, the disease itself has instilled a sense of fear among front line workers, people with COVID 19 and population at large, this effect has been amplified by the overuse of social media which has led to an Infodemic (Orso et al., 2020; Vaezi & Javanmard, 2019). The fear due to disease could affect the population in general whereas it can have a precipitating effect of mental status of people with existing mental health conditions. The response to pandemic has led to a complete or partial restriction of movement in many countries. ‘Lockdown’, closure of educational centers and workplaces can have a significant impact on mental health due to changes in daily routine, social isolation in population, predominantly in children and older people, and people with existing mental health conditions. Abundance evidence is available to suggest that excessive use of social media has a significant impact on mental health (Ahmad & Murad, 2020; Gao et al., 2020; Ni et al., 2020). COVID 19 pandemic and restrictions imposed because of it had led to a surge in screen time as well as social media exposure. Closure of hospitals for non-essential services, in order to meet the surge capacity and halt the disease transmission, has caused serious disruption in routine health care services in many countries including India (Banerjee, 2020; How covid-19 response disrupted health services in rural India, 2020; COVID-19 significantly impacts health services for noncommunicable diseases, 2020). Non-provision of essential services may have a serious impact of older people, people with mental health conditions and chronic conditions. Whereas, a change in working pattern and increase perception of risk of disease can lead to anxiety, depression, stress and burnout among health care workers. Health care workers are also at risk of moral injury apart from recognized mental health conditions such as depression or post-traumatic stress disorder. Moral injury has been explained as a term that originated in the military and can be defined as the psychological distress that results from actions, or the lack of them, which violate someone’s moral or ethical code. People with moral injury are likely to experience negative thoughts about themselves or others as well as intense feelings of shame, guilt or disgust. As we have searched multiple portals to gather information and have also compiled results from various research papers available, the paper gives us a detailed overview of the mental health issues during COVID-19 and its responses, however a limitation of this review could be that, the information regarding data availability or sampling frame was not possible to be explored from every source of information, thus there could be a question about its generalizability to the population.

We propose a multi-pronged multi-stakeholder approach based on the experiences and evidence available from India and different countries. International and national organizations, governments, non-governmental organizations, health professionals’ groups and self-help groups are important stakeholders in providing interventions. Multi-pronged approach should comprise of a helpline number for easy access to mental health support, strict control over misinformation in social media, provision of continuum of care services at all level, financial and employment security to vulnerable groups, regulatory and legal provisions against discrimination and stigma of health care workers and other forces involved in pandemic response. We also suggest a framework for interventions and recommendations for specific population groups based on the possible reasons associated with their enhanced risk to mental health problems (Table 3).

**Table 3.** Probable reasons of mental health issues and recommended intervention strategies for COVID-19 related mental health problems among different vulnerable population groups.

Vulnerable Population groups	Probable reasons of mental health issues during COVID-19 pandemic	Recommended intervention strategies for COVID-19 related mental health problems
Children	• Changes in the day to day routine • Closure of schools and restriction of outdoor recreational activities Not meeting their friends and peer	• Reducing screen-time to avoid negative news but providing clear information Engaging in creative and mentally stimulating indoor activities • Managing a child’s anxiety by identifying their emotional needs • Making ways to keep in touch with their friends Making a home learning routine
Geriatric Population	• The elderly have underlying comorbid conditions causing fear and anxiety of the consequences of getting infected • Difficulty in day to day activities for those living alone • Sense of social isolation due to lockdown Difficulty in availing online or telemedicine services for healthcare due to challenges in handling smartphones or computers, thus hampering their routine healthcare	• Giving out clear, concise and necessary information in a respectful way. • Assurance and assistance to the more vulnerable. • Engage family members and support workers carefully deal with mental health issues • Connecting with loved ones living away Engaging in recreational activities. • Spending time off the news Ensuring enough medications for those in need to address any insecurities.
Migrant workers	• Less familiar in their new environment in which they temporarily live • Concerned about their families who are living elsewhere Financial and economic loss for the daily wage workers	• Treating every migrant worker with dignity, respect, empathy and compassion individually without generalisation Emphasising the need to stay away from their families and providing assurance of mental and physical support • Ensuring respectful economic assistance and assurance Constant systematic assurance, effective counselling and providing the basic needs
Frontline workers	• Chances of contracting the disease while caring for the people. • Fear of transmitting the disease to their family members. • Extensive work hours as the burden on the healthcare system increases. Stress due to moral injury	• Ensuring ergonomic environment by providing proper and adequate protective gear • Providing incentives to them and their families for risking their lives to take care of others • Identifying their situation of moral injury and addressing them. • Ensuring workplace respect and safety Recognising their selfless efforts and rewarding them appropriately
People with COVID-19, contacts, survivors, family members	• Complete isolation from near and dear ones • The feeling of being the cause of transmitting the disease to others Discrimination causing emotional trauma	• Addressing the grief and trauma faced by people with COVID19 and their family Creating self-help platforms • Helping them cope with emotional loss if they have lost a family or friend to the grave pandemic Recognizing the survivors and providing them the mental and physical comfort at their isolation sites or hospitals
People with existing mental illnesses	• Isolation, Quarantine and being confined at home is a trigger factor • Continuum of care may be affected as tele-counselling sessions may not be as effective as face to face sessions. Non-availability of entertainment factors A change in the daily routine of people with pre-existing mental illness	• Providing access to treatment through telemedicine consultations and video consultations. • Adequately modifying their counselling sessions to help them cope with the pandemic along with their already existing illness. Involving Family members in their care and attention.

## Conclusion

While the health system struggles to save millions of lives daily, there is probably a risk of a looming pandemic of hidden mental health issues which has a huge potential of shattering the existing mental health infrastructure. To handle the aftermath of the COVID-19 pandemic, the mental health of the people needs to be handled hand in hand and given equal importance along with other strategies to manage and control the disease and the pandemic at large. There is a definite need for specialized psychological intervention and proper and consistent risk communication and crisis communication. An updated, timely, uncomplicated guidelines should be put forth in order to avoid confusion and anxiety among the people. Hence, keeping a positive approach, effective communication strategies and understanding the problem statement, will help in dealing with the mental health issues faced by the world in this hour of crisis. The recommended intervention strategies should therefore be vulnerable group specific and further cause or risk factor specific also. Developing need-based interventions with proper risk communication strategies and keeping at par with the evolving epidemiology of COVID-19 would be instrumental in guiding the planning and prioritization of mental health care resources so that the mental health of most vulnerable groups is well served.
